# Safety, feasibility, and comfort of hepatic angiography and transarterial intervention with radial access for hepatocellular carcinoma

**DOI:** 10.1002/jgh3.12628

**Published:** 2021-07-30

**Authors:** Hidenori Toyoda, Satoshi Yasuda, Shohei Shiota, Shohei Chatani, Ryota Tsukii, Hirofumi Kitagawa, Tomohisa Fukushima, Shohei Urasaki, Takashi Kumada

**Affiliations:** ^1^ Department of Gastroenterology Ogaki Municipal Hospital Ogaki Japan; ^2^ Department of Radiological Diagnosis and Interventional Radiology Aichi Cancer Center Hospital Nagoya Japan; ^3^ Department of Medical Technology Ogaki Municipal Hospital Ogaki Japan; ^4^ Department of Nursing Gifu Kyoritsu University Ogaki Japan

**Keywords:** comfort, feasibility, HCC, hepatic angiography, radial access, safety

## Abstract

**Background and Aim:**

Hepatic angiography procedures such as transarterial chemoembolization (TACE) are essential procedures for managing patients with hepatocellular carcinoma (HCC), and are usually performed with femoral access. However, femoral access causes patient discomfort and may be associated with the risk of hematoma or pseudoaneurysm at puncture site. We evaluated the safety, feasibility, and patient comfort of hepatic angiography procedures performed with radial access.

**Methods:**

In this single‐institution, retrospective, time‐frame study, a total of 206 patients who underwent hepatic angiography procedures with radial access, which were first used on October 2017 at our institution, were compared with 240 patients who underwent the same procedures with femoral access before this period. Several measures were assessed, including procedure time and safety. In addition, a questionnaire was used to compare the access types regarding procedure‐associated discomfort.

**Results:**

Hepatic angiography procedures performed with radial access, including TACE, were completed in all patients without complications. The procedure time was comparable between radial access and femoral access. Most patients preferred radial to femoral access. Patients taking anticoagulants were able to complete the procedures without discontinuing these drugs.

**Conclusions:**

Hepatic angiography procedures with radial access resulted in less discomfort than those with femoral access, and the two approaches showed similar feasibility and safety. Radial access can be introduced as a routine technique for hepatic angiography procedures.

## Graphical abstract

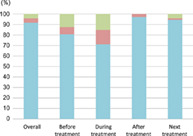

## Introduction

Hepatic angiography procedures are indispensable techniques for the management of patients with hepatocellular carcinoma (HCC). Transarterial chemoembolization (TACE) is one of the main treatment modalities for HCC. In addition, the information from computed tomography (CT) images during arterial portography (CTAP) or hepatic arteriography (CTHA) is sometimes helpful to confirm the diagnosis of hepatic nodules that do not show typical imaging features of HCC.^1^, ^2^ In such a case, the use of hepatic angiography is necessary to obtain images.

Hepatic angiography procedures usually involve femoral artery access in the inguinal region. By contrast, current cardiac angiographic procedures such as coronary angiography or percutaneous coronary intervention are usually performed with access through the radial artery at the wrist. Recently, abdominal angiography procedures, including hepatic angiography and TACE, have been attempted with radial access,^3^, ^4^ and this approach is routinely used in some countries. Although hepatic angiography and TACE through radial access can merit by reducing patient discomfort or the risk of bleeding complications from the puncture site, it has been underused due to concerns regarding prolonged procedure time, prohibitive vascular anatomic complexity, or distance from the access site. Therefore, in this study, we evaluated the safety and feasibility of hepatic angiography procedures using radial access by comparing them with conventional femoral access.

## Patients and methods

### Study patients

Hepatic angiography and TACE with radial artery access were introduced at Ogaki Municipal Hospital in October 2017. Patients who underwent these procedures between October 2017 and March 2020 were studied retrospectively. During the study period, all patients were principally considered to undergo hepatic angiography and TACE with radial artery access. A total of 98 patients were diagnosed as having initial HCC and 76 of these patients underwent hepatic angiography or TACE during the study period. In addition, 133 patients underwent hepatic angiography or TACE for recurrent HCC. All patients underwent Allen's test before the procedure to confirm the presence of ulnar arterial flow, because the placement of a sheath or catheter into the radial artery in the absence of ulnar arterial flow may induce ischemia of the hand. Allen's test was negative in one patient, who was advised to undergo hepatic angiography with access through the femoral artery. In addition, two patients who previously underwent TACE with femoral access refused that with radial access and excluded. Therefore, we analyzed 206 patients as a radial access group. Femoral access group consisted of patients who underwent hepatic angiography examination or TACE with vascular access through the femoral artery between April 2015 and September 2017.

Prior to hepatic angiography, HCC was diagnosed in all patients based on outpatient‐based dynamic CT or magnetic resonance imaging (MRI) with early arterial enhancement and delayed washout. The diagnosis of HCC was further confirmed by imaging findings from CTAP and CTHA in some cases that did not show typical imaging features of HCC by outpatient‐based imaging examinations.

The study protocol complied with the Helsinki Declaration and was approved by the institutional review board. The requirement of informed consent was waived, as we used only de‐identified data collected from medical records.

### Hepatic angiography procedures with access through the radial artery and the femoral artery

**Figure 1:**
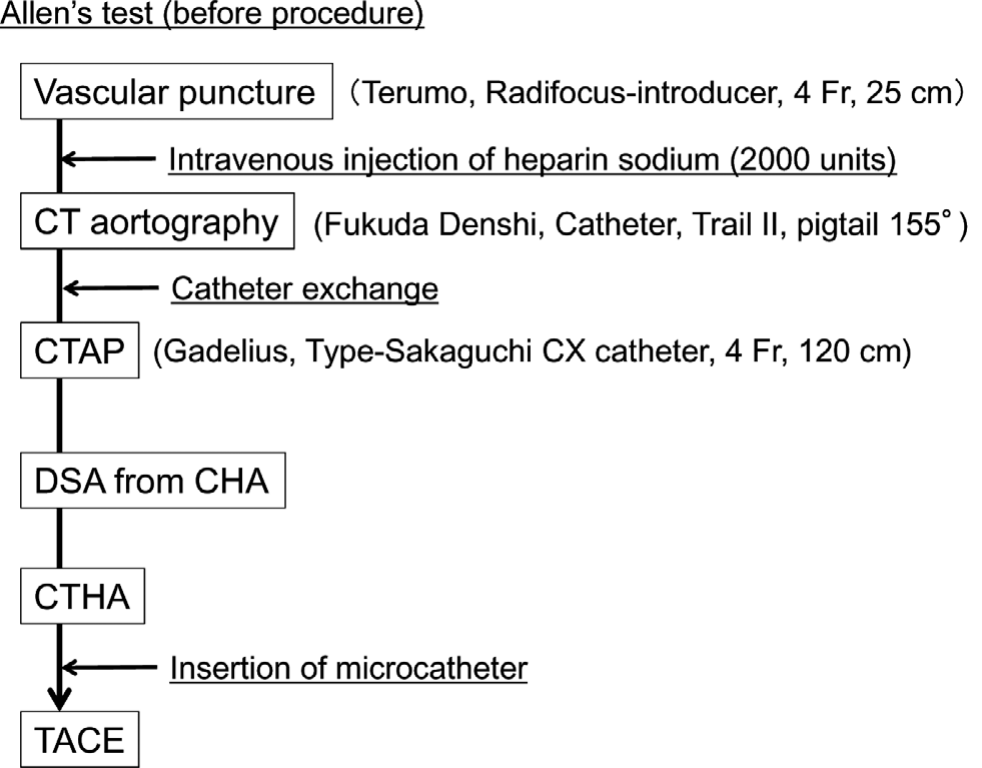
Flowchart of hepatic angiography procedures with radial access.

Angiography was performed by five of the authors (HT, SY, SS, SC, and RT) with 6–20 years of experience of abdominal angiography (Fig. 1). The entire procedure was performed with an angio‐CT system equipped with 64‐multidetector‐row CT (Aquilion CX; Toshiba Medical Systems, Tokyo, Japan) and angiography units with DSA equipment and a C‐arm (Infinix Celeve‐i INFX 8000C; Toshiba Medical Systems). Each patient was placed in supine position with his or her legs oriented toward the CT machine (Fig. S1). The left wrist and ante‐brachial area were disinfected, the left radial artery was punctured with local anesthesia, and then a 4‐Fr long sheath (Radifocus‐introducer RR‐AF4D25H, 4 Fr, 25 cm Terumo, Tokyo, Japan) was placed (Fig. S2). After placement of the sheath, 2000 units of heparin sodium was injected intravenously to prevent the formation of microthrombi around the inserted catheter, as these may cause cerebrovascular emboli. A pigtail catheter (Fukuda catheter for contrast enhancement, Trail II, pigtail 155°, P155, 4 Fr, 110 cm, Fukuda Denshi, Tokyo, Japan) with a guidewire (Radifocus‐guidewire, RF‐HA 35263, 0.035 inch, 260 cm, Terumo) was then inserted via the sheath and was advanced into the radial, brachial, and subclavian arteries to the aortic arch with the guidewire preceding the tip of the catheter. The catheter tip was advanced into the descending aorta to the level of the carina. Dynamic contrast‐enhanced CT aortography was then performed with the injection of 90 mL of nonionic contrast medium (Iopamiron 300; Bayer Yakuhin, Osaka, Japan) diluted with saline at 1:2 ratio by volume, which was injected at a rate of 9 mL/s.

After CT aortography, the catheter was exchanged. The pigtail catheter was withdrawn and the guidewire was left in the descending aorta, then an abdominal catheter used for the contrast enhancement of abdominal arteries (Type—Sakaguchi CX catheter, 4 Fr, 115 cm, Gadelius, Tokyo, Japan) was inserted into the superior mesenteric artery (SMA) and after that common hepatic artery (CHA) through the celiac axis. In all patients, we first performed CTAP examination with the tip of the catheter in the SMA. After CTAP, digital DSA of the hepatic artery and CTHA were performed with the tip of the catheter in the CHA (Appendix S1). Patients in whom TACE was selected for the treatment of HCC subsequently underwent the TACE procedure. Target‐feeding arteries were first identified based on DSA and 3D CT aortography images.^5^ A microcatheter (Veloute, VEL150‐16SR; 1.7 Fr, 150 cm, Asahi‐Intecc, Tokyo, Japan) was inserted through the abdominal catheter into the identified target arteries with 0.016‐inch guidewire (Meister, AMS‐180‐16WAR; 0.016 inch, 180 cm, Asahi‐Intecc). There were no differences in TACE procedure between the access through radial artery and femoral artery (Appendix S1).

After the hepatic angiography procedure, the abdominal catheter was withdrawn with the guidewire protruding beyond the catheter tip to prevent cerebrovascular embolism during withdrawal. After withdrawal of the sheath, a wristband (T‐R band, XX‐RF06; Terumo) was attached (Fig. S3) to prevent bleeding from puncture site. The compression pressure was fixed at 240 mmHg at attachment and gradually reduced, 160 mmHg at 20 min and 100 mmHg at 1 h after attachment. The band was removed 4 h after sheath withdrawal.

In femoral access group, angiography was performed using the same angiography system. A 4‐Fr long sheath (Radifocus‐introducer RR‐A40K25AK, 4 Fr, 25 cm Terumo) was inserted from the right common femoral artery into abdominal aorta. A pigtail catheter (Medikit catheter for contrast enhancement, PTA‐K, pigtail, 3.3 Fr, 90 cm, Medikit, Tokyo, Japan) was then inserted via the sheath and was advanced into the descending aorta to the level of the carina for CT aortography. After that, the catheter was changed to an abdominal catheter (GHC‐A catheter, 4.2 Fr, 85 cm, or Cobra catheter, 4.2 Fr, 70 cm, Hanaco Medical, Saitama, Japan) and CTAP, DSA, and CTHA were performed with the tip of the catheter at the same position as radial access in all patients. After the identification of target arteries by DSA and 3D CT, TACE was performed through the abdominal catheter inserting microcatheter (Veloute, VEL125‐16S; 1.7 Fr, 125 cm, Asahi‐Intecc) into target arteries with 0.016‐inch guidewire (Meister, AMS‐180‐16WAR; 0.016 inch, 180 cm, Asahi‐Intecc). After withdrawal of the sheath, 15 min of manual compression was applied to the femoral access site. Further compression was performed when hemostasis could not be achieved. Then, patients were monitored in bed rest with immobilization of the affected leg for 4 h.

### Comparison of procedure time by access routes

To evaluate the feasibility of hepatic angiography procedures with radial access *versus* femoral access, we compared two access types in terms of the length of time from the placement of the arterial sheath to CT aortography, and from the placement of the sheath to digital subtraction arteriography (DSA) performed in the CHA after CTAP. The data on the time of the placement of the sheath, CT aortography, and DSA from CHA were collected from electronic medical records of respective patients.

### Questionnaire about discomfort associated with hepatic angiography procedures

**Figure 2:**
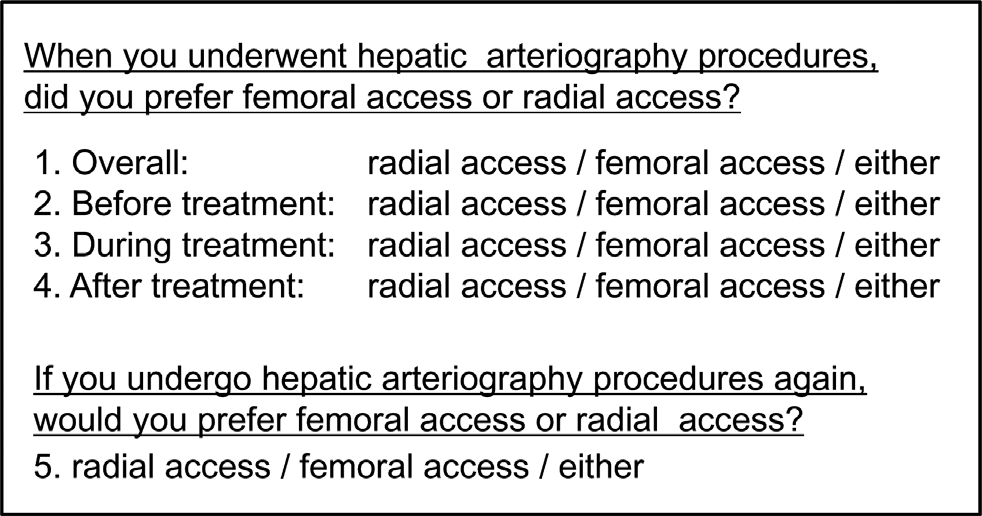
Questionnaire for patients who experienced hepatic angiography procedures with both radial access and femoral access comparing the two types of access in terms of satisfaction and discomfort.

All 73 patients who had experienced hepatic angiography procedures with femoral access and subsequently underwent repeat hepatic angiography procedures with radial access (i.e. patients who experienced hepatic angiography procedures with both femoral and radial access) were asked on the day following the radial procedure to complete a questionnaire comparing the two access types (Fig. 2). They were requested to simply report their preferences between femoral and radial access based on their experience and level of discomfort, overall as well as before, during, and after the procedure.

### Statistical analysis

Categorical variables are expressed as numbers and percentages, and continuous variables are expressed as medians and interquartile ranges. Differences in percentages between groups were analyzed with the chi‐square test. Differences in quantitative values were analyzed by the Mann–Whitney *U* test. Change in quantitative values was analyzed with Jonckheere–Terpstra test. Statistical analysis was performed using JMP statistical software, version 11.0 (SAS Institute, Cary, NC, USA). All *P* values were derived from two‐tailed tests, with *P* < 0.05 accepted as statistically significant.

## Results

### Characteristics of study patients and hepatic angiography procedure

Table 1 shows the backgrounds of the study patients. The percentage of male patients was higher in patients who underwent hepatic angiography procedures with radial access than in patients with femoral access. Nonviral HCC was more frequent in patients who underwent hepatic angiography with radial access, and fewer patients with radial access subsequently underwent TACE. More than 60% of patients had an experience to undergo hepatic angiography in both groups, and 57 of 131 patients underwent more than once through radial access.

**Table 1:** Backgrounds of study patients

	Femoral access (*n* = 240)	Radial access (*n* = 206)	*P* value
Age (years)	75 (68–79)	72.5 (68–78)	0.1347
Gender (female/male)	58 (24.2)/182 (75.8)	34 (16.5)/172 (83.5)	0.0471
Etiology (HBV/HCV/nonviral)	27 (11.3)/129 (53.8)/84 (35.0)	33 (16.0)/78 (37.9)/95 (46.1)	0.0035
Platelet count (10^3^/μL)	127 (90–166)	138 (96–182)	0.0679
Prothrombin time (%)	83.5 (73.0–94.0)	85.0 (73.0–95.0)	0.4903
Past experience of HA (no/yes)	71 (29.6)/169 (70.4)	75 (36.4)/131 (63.6)	0.1303
Procedure (arteriography alone/TACE)	79 (32.9)/161 (67.1)	87 (42.2)/119 (57.8)	0.0494

Values in parentheses are interquartile ranges or percentages.

HA, hepatic arteriography procedure; HBV, hepatitis B virus infection; HCV, hepatitis C virus infection; TACE, transarterial chemoembolization.

Hepatic angiography and TACE were successfully performed in all patients. No patients experienced the switch of access site from radial access to femoral one. Marked meandering of the cubital/brachial artery was observed in three patients but the artery was straightened by the insertion of the guidewire (Fig. S4). The operator did not experience difficulty when using a microcatheter and microguidewire to perform TACE. Extrahepatic feeding arteries were found in 38 patients; most of these involved the right inferior phrenic artery (*n* = 35) and others involved the omental artery (*n* = 1) or right adrenal artery (*n* = 2). All feeding arteries were appropriately embolized. When the wristband was removed, bleeding from the puncture site was observed in five patients but it stopped after an additional 3 h of compression, with no re‐bleeding. No hematoma was observed at the site of puncture.

### Time required for angiography procedures

**Figure 3:**
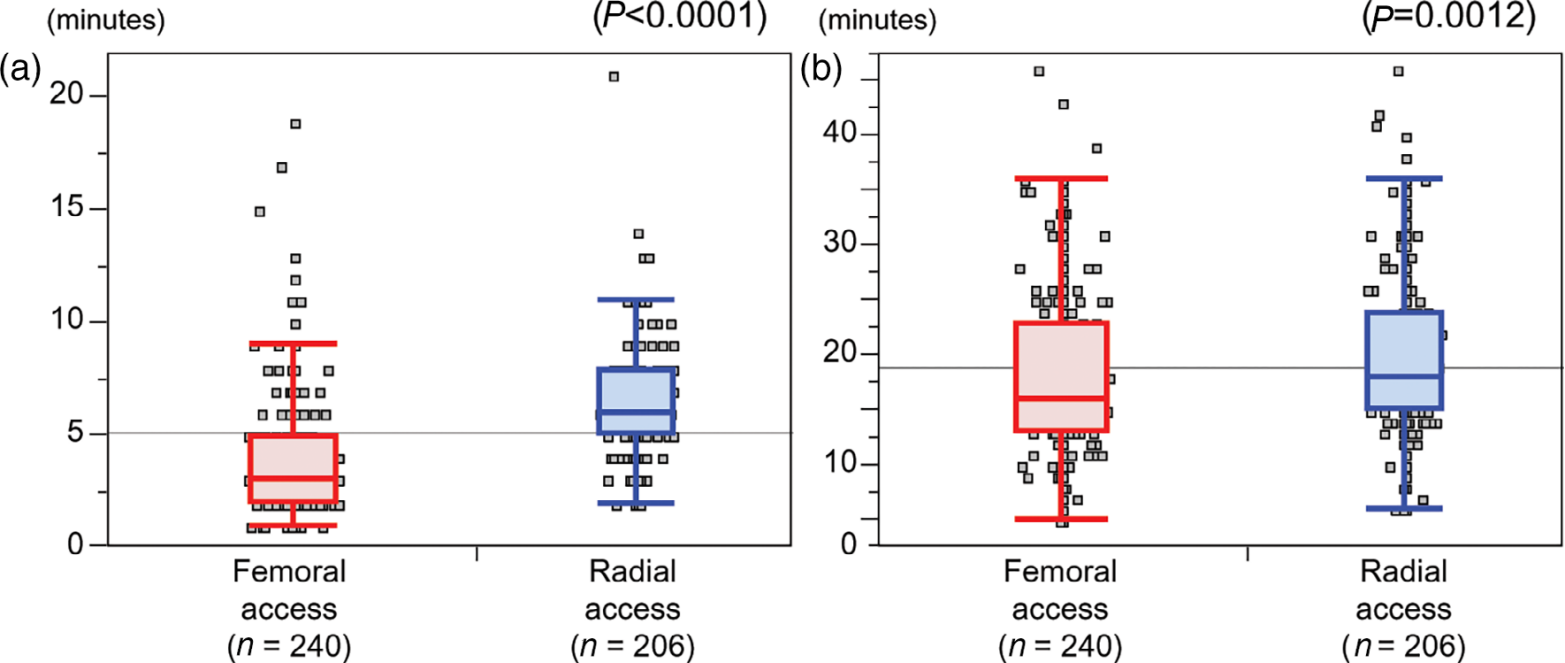
Comparisons between femoral a and radial access procedures in terms of time required for catheter insertion from sheath placement in the radial of femoral artery (a) to computed tomography aortography and (b) to digital subtraction arteriography from the common hepatic artery.

Figure 3 shows the times required from the placement of the sheath in the radial or femoral artery to CT aortography (A) and to DSA from the CHA (B). Both median times were shorter in patients whose procedures were performed with femoral access rather than radial access. The median time to CT aortography was 3.0 min with femoral access and 6.0 min with radial access (*P* < 0.0001), and the median time to DSA was 16.0 min with femoral access and 18.0 min with radial access (*P* = 0.0012). In contrast, there was no difference in the total fluoroscopic time between radial access and femoral access in patients who underwent only hepatic angiography (median time: 9.55 min with radial access *versus* 9.6 min with femoral access, *P* = 0.2489). When compared according to patient age, no differences were observed in procedure time between patients aged <75 years (*n* = 229) and patients aged ≥75 years (*n* = 217) in both femoral access and radial access. The median time to CT aortography in patients aged <75 years and ≥75 years were both 3.0 min (*P* = 0.4519) with femoral access and both 6.0 min (*P* = 0.8507) with radial access. The median time to DSA in patients aged <75 years and ≥75 years were both 16.0 min (*P* = 0.6186) with femoral access and both 18.0 min (*P* = 0.4384) with radial access.

**Figure 4:**
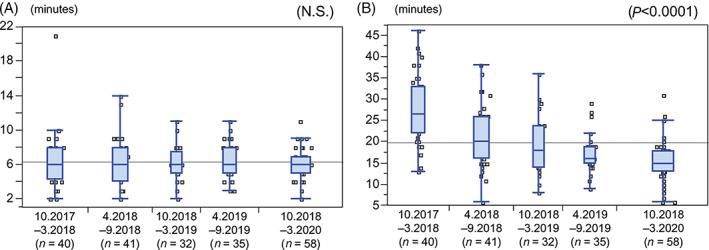
Changes in time required for catheter insertion from sheath placement in the artery (a) to computed tomography aortography and (b) to digital subtraction arteriography from the common hepatic artery, according to time periods since our institution began performing hepatic angiography procedures with radial access.

Figure 4 indicates how the duration of hepatic arteriography procedures with radial access changed over the course of 30 months (five 6‐month periods), beginning when radial access was first used at our institution; in other words, the figure demonstrates the operator learning curve for this technique. Specifically, for each period, the figure shows the times required from the placement of the sheath in the radial artery to CT aortography (A), and from the placement of the sheath to DSA from the CHA (B). Whereas the median times to CT aortography remained constant throughout the 30‐month period, the median time to DSA decreased (*P* < 0.0001). In the last three periods (between October 2018 and March 2020, *n* = 124), the median time to DSA with radial access did not differ from that with femoral access (both median, 16.0 min, *P* = 0.8314).

### Patient satisfaction by procedure access sites

**Figure 5:**
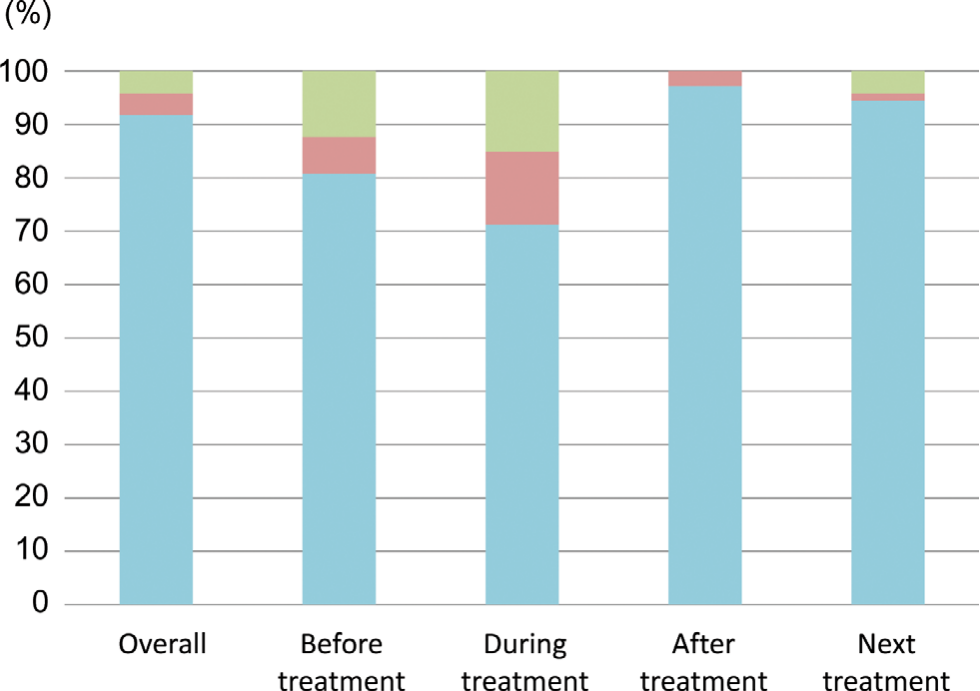
Results of the questionnaire comparing preferences between radial access and femoral access. 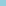, radial; 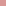, femoral; 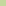, either.

Figure 5 shows patient preferences by hepatic angiography procedure access site. The majority of patients preferred radial access, overall as well as before, during, and after treatment. In particular, 71 of 73 patients (97.3%) reported a preference for radial access after hepatic angiography procedures and 69 of 73 patients (94.5%) preferred radial access if they were to undergo hepatic angiography procedures again. Patient preference of radial access did not differ based on time period of the study, patient demographics, or with or without TACE. Furthermore, all patients who underwent hepatic angiography procedure through radial access more than once again preferred radial access for future hepatic angiography.

No patients were required sedation for their discomfort during or after the procedure. Whereas 46 of 240 patients (19.2%) of femoral access group were prescribed pain medication, no patients of radial access group required it.

### Angiography procedures on anticoagulant drugs

Thirteen patients of radial access group underwent hepatic angiography procedures without stopping their pre‐existing anticoagulant therapy due to a high risk of vascular embolization (warfarin in eight patients, Edoxaban Tosilate Hydrate in one patient, and Clopidogrel Sulfate in four patients). In these patients, wristband compression was extended up to 24 h. No problems were observed on their banded hand, including ischemia and pain, and no bleeding occurred from the puncture site after compression was stopped and the band was removed.

## Discussion

The degree of patient discomfort is an important factor that should be considered for every medical procedures. Angiography with femoral artery access requires more than 3 or 4 h of continuous immobilization often with compression at the inguinal area puncture site in order to prevent bleeding after withdrawal of the catheters and sheath. This requires patients to continuously maintain a supine position on a bed and to minimize body movement, which can be highly uncomfortable,^6^, ^7^ frequently associated with lumbar pain. In particular, patients often feel nauseous just after TACE, and avoiding movement worsens this sensation. Also, femoral access may increase the risk of hematoma or pseudoaneurysm at puncture site.^8^ For these reasons, radial access can be an option for hepatic angiography or TACE. However, hepatic angiography is rarely performed with radial access in Japan.

It this study, we used radial access to perform hepatic angiography procedures, that is, hepatic angiography evaluation of liver tumors including CTAP and CTHA, and TACE. Although vascular intervention with radial access is often thought to increase the risk of cerebrovascular complications, previous large studies showed no change in the incidence of complications.^8^, ^9^ Indeed, in our preliminary experience of 206 patients, no cerebrovascular complications occurred.

The time required for an entire TACE procedure varies widely, based, for instance, on the anatomy of target‐feeding arteries, since this affects the difficulty of inserting microcatheter, and on the number of HCC tumors that are treated. Therefore, in this preliminary study, we sought to compare the feasibility of radial access and femoral access by comparing the time from the placement of the sheath in the artery to CT aortography, and from the placement of the sheath to DSA from the CHA that was performed after CTAP from SMA in all patients. In our institution, CT aortography is routinely performed before CTAP, CTHA, or DSA. Because outpatient‐based cross‐sectional imaging study for liver tumor is MRI, dynamic enhanced CT was rarely performed before angiography in our institution. Therefore, CT aortography is helpful to obtain the information of extrahepatic feeding to HCC. In addition, the information from CTAP or CTHA is often helpful to confirm the diagnosis of hepatic nodules that do not show typical imaging features of HCC.^1^, ^2^

The time from sheath placement to CT aortography was significantly longer in patients with radial access than those with femoral access. This is partly because with radial access, the pigtail catheter used in aortography passes through the bifurcation of the cerebral artery (left internal carotid artery and left vertebral artery) when it is inserted into the descending aorta, and the insertion should be performed slowly and very carefully. By contrast, there were several patients with femoral access who required a very long time to CT aortography or DSA, despite the shorter median times (Figs 3 and 4). We sometimes experience patients with extreme meandering of the abdominal aorta (Fig. S5), probably due to atherosclerosis, in whom insertion of the catheter through the abdominal aorta or catheterization to the SMA or CHA is very difficult. In such cases, radial access may be more feasible. By contrast, there were no differences in procedure times based on patient age (<75 years *vs* ≥75 years) in both femoral access and radial access. Therefore, arteriosclerosis associated with aging may not have strong influences on the procedure time regardless of access sites.

With radial access, the time required from sheath placement to CT aortography was constant throughout the study period. By contrast, the time from sheath placement to DSA decreased markedly. The time to DSA did not differ between radial access and femoral access at 1 year after the start of the radial access procedure, indicating that 1 year of experience is sufficient to master this technique.

One advantage of using radial access to perform hepatic arteriography procedures is reduced patient discomfort, especially after the procedures are complete. Indeed, patients can sit and walk almost immediately afterward. Thus, it is unsurprising that most patients who underwent procedures with both radial access and femoral access preferred the former. In particular, most patients preferred receiving potential repeated hepatic angiography procedure in the future through radial access. This will be important because HCC recurs frequently and TACE is often necessary to be repeated several times. In addition, angiography procedures, including TACE, can be performed without the need to discontinue anticoagulant drugs with radial access, thus avoiding an increased risk of vascular embolization associated with temporary discontinuation of anticoagulant drugs.

A disadvantage of radial access is that it can preclude the use of TACE for embolization of some arteries. When stenosis of the celiac axis or CHA is present, catheterization of the proper hepatic artery should be performed via the SMA, pancreaticoduodenal arcade, and gastroduodenal artery; however, this is difficult with radial access due to the insufficient length of the microcatheter and microguidewire. When HCC is fed by the right internal thoracic artery, it is difficult to catheterize it from the right subclavian artery with left radial access.

There are several limitations to this study. The study used a retrospective design, and the two patient groups, that is, those with radial access *versus* femoral access, were not controlled. In addition, the enrollment periods of the two groups were different, although the same angiographic apparatus was used. Also, the questionnaire that compared radial access and femoral access was always administered after hepatic angiography procedures performed with radial access, which may have resulted in bias. We observed no difference in the feasibility of TACE between femoral and radial access, but this should be confirmed by further prospective studies. Also, it will be necessary to investigate the long‐term incidence of complications with a larger number of cases in order to confirm the safety of this procedure, since this study evaluated only 206 patients over a 2.5‐year period.

In conclusion, this preliminary study of hepatic angiography procedures with radial access revealed the safety and feasibility of this approach. Angiographic examination procedures and TACE showed similar feasibility with radial access and femoral access. By contrast, compared with femoral access, radial access was associated with less discomfort and higher patient satisfaction.

## Supporting information

**Figure S1** The angiography and CT apparatus used for angiography and the standard patient positioning during hepatic angiography procedures with radial access.

**Figure S2** The preparation and puncture of the left radial artery.

**Figure S3** The wristband that compresses the puncture site to achieve hemostasis after sheath withdrawal.

**Figure S4** Straitening of a markedly meandering cubital/brachial artery by insertion of a guidewire.

**Figure S5** Marked meandering of the abdominal aorta depicted using 3D‐CT images.

**Appendix S1**: Supporting information.
