# Supplementary figures and images for: Safety, feasibility, and comfort of hepatic angiography and transarterial intervention with radial access for hepatocellular carcinoma
Source: JGH Open. 2021 Jul 30;5(9):1041–6. doi: 10.1002/jgh3.12628 (PMC8454468; doi:10.1002/jgh3.12628)

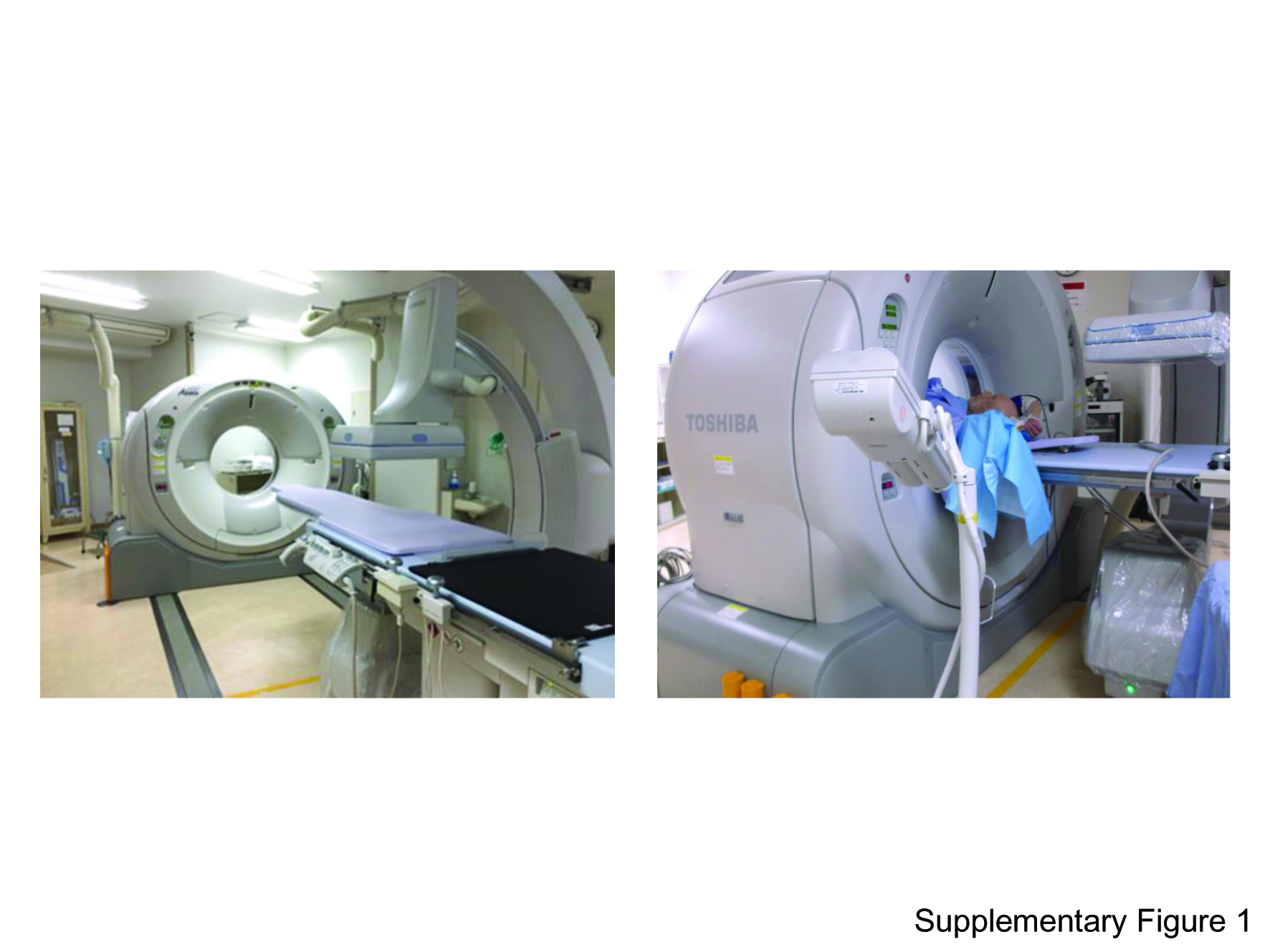

Supplement: Supplementary file 1 — Figure S1 The angiography and CT apparatus used for angiography and the standard patient positioning during hepatic angiography procedures with radial access. [file JGH3-5-1041-s003.tif]

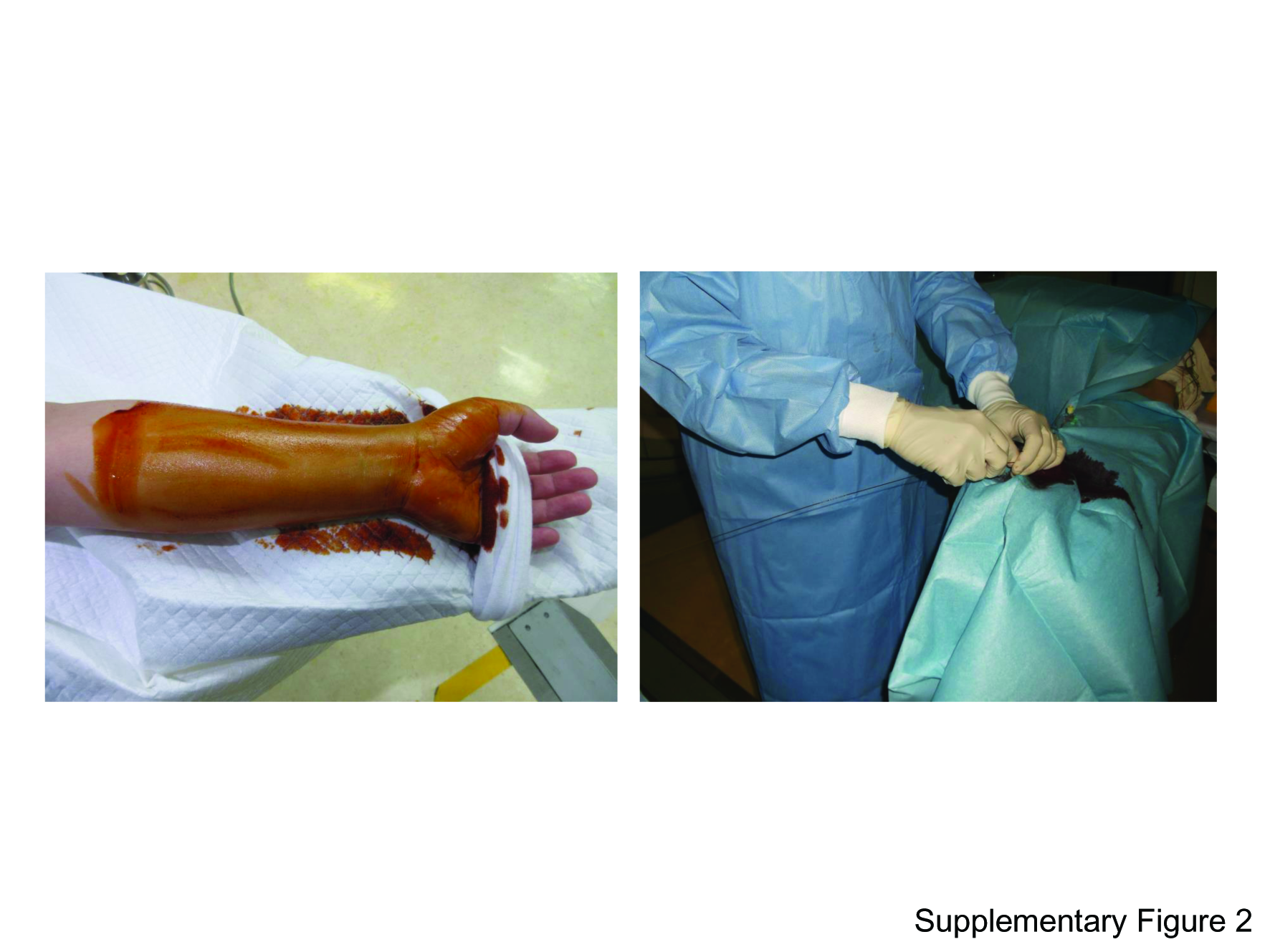

Supplement: Supplementary file 2 — Figure S2 The preparation and puncture of the left radial artery. [file JGH3-5-1041-s001.tif]

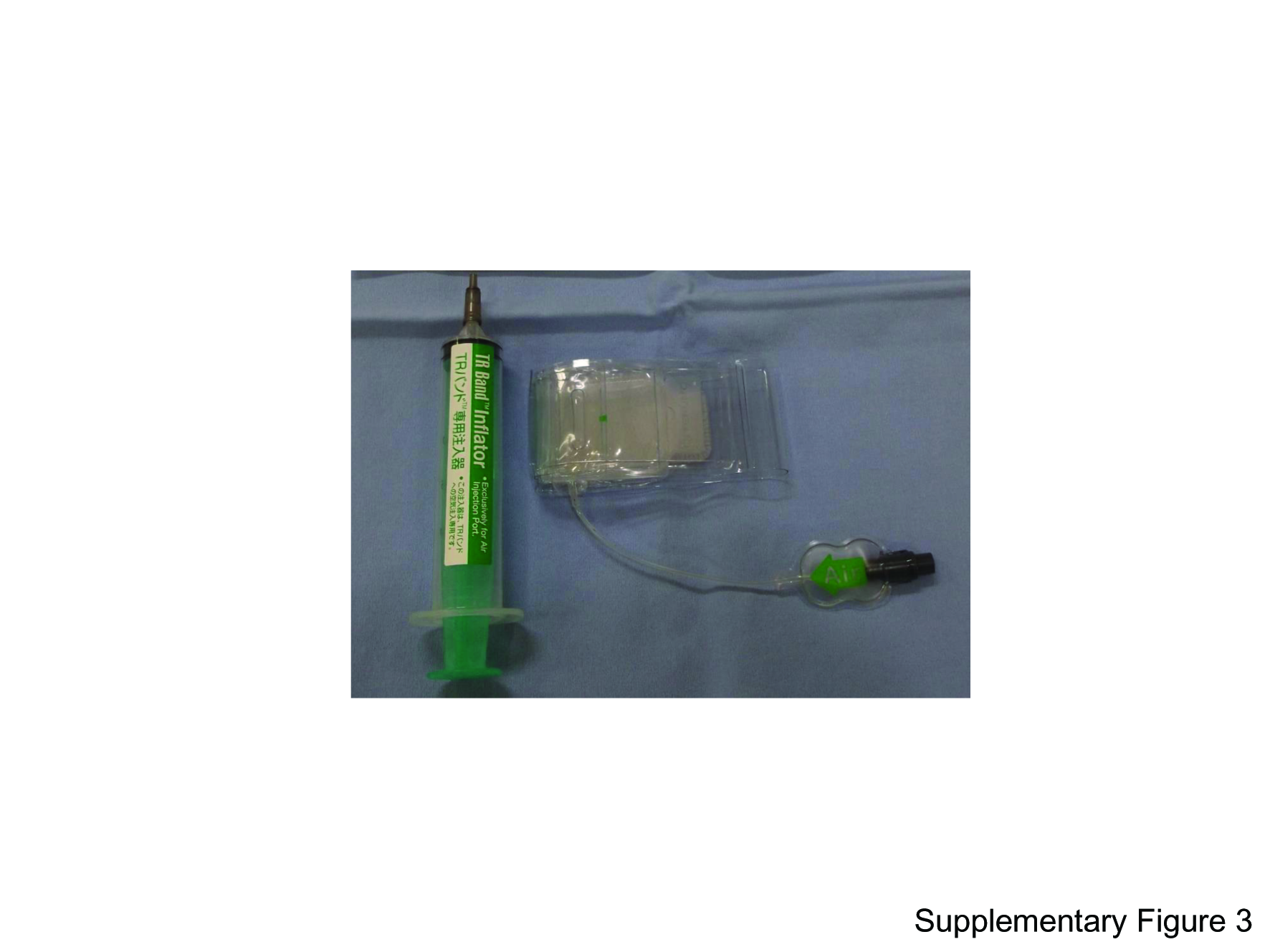

Supplement: Supplementary file 3 — Figure S3 The wristband that compresses the puncture site to achieve hemostasis after sheath withdrawal. [file JGH3-5-1041-s004.tif]

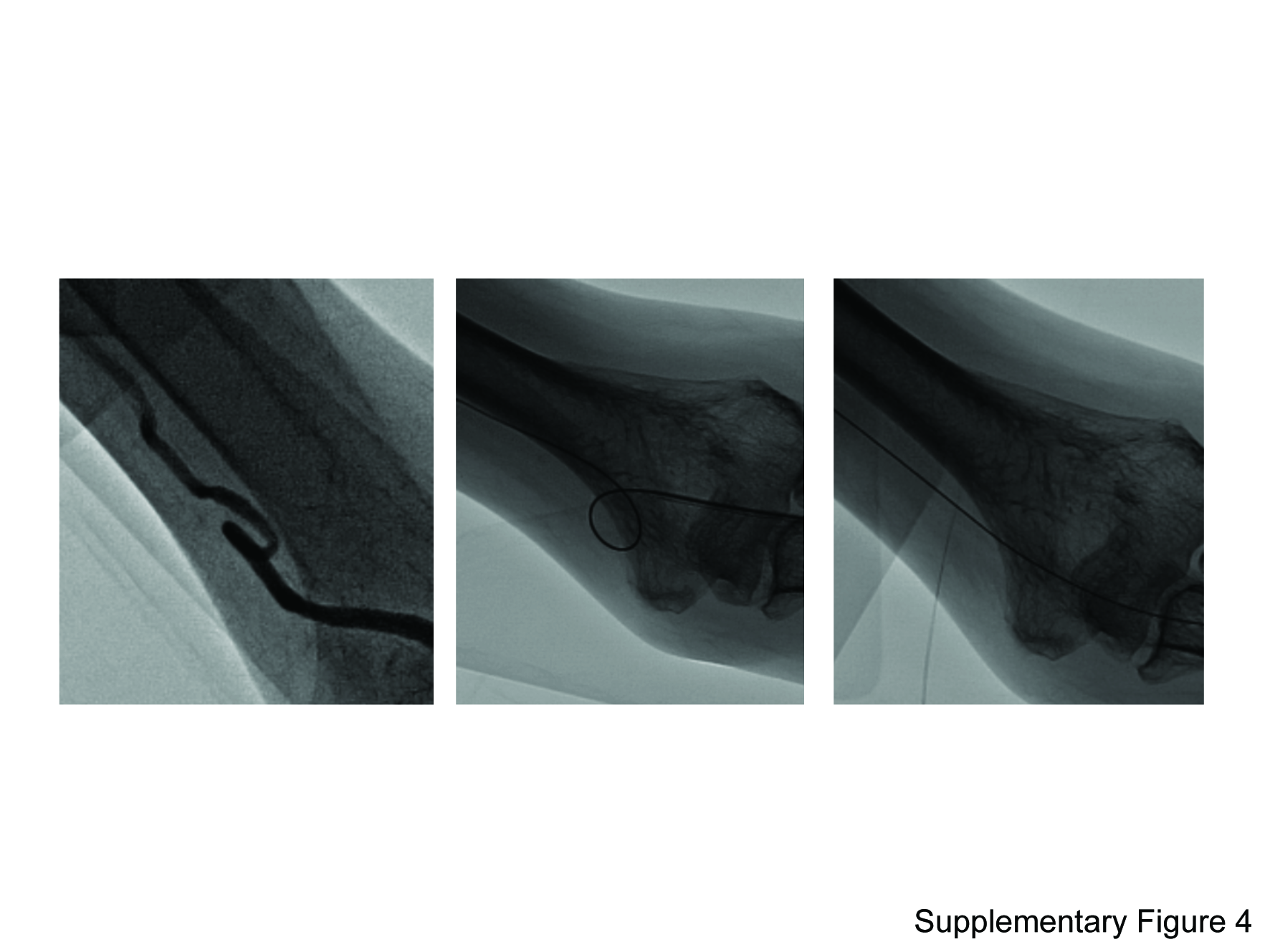

Supplement: Supplementary file 4 — Figure S4 Straitening of a markedly meandering cubital/brachial artery by insertion of a guidewire. [file JGH3-5-1041-s005.tif]

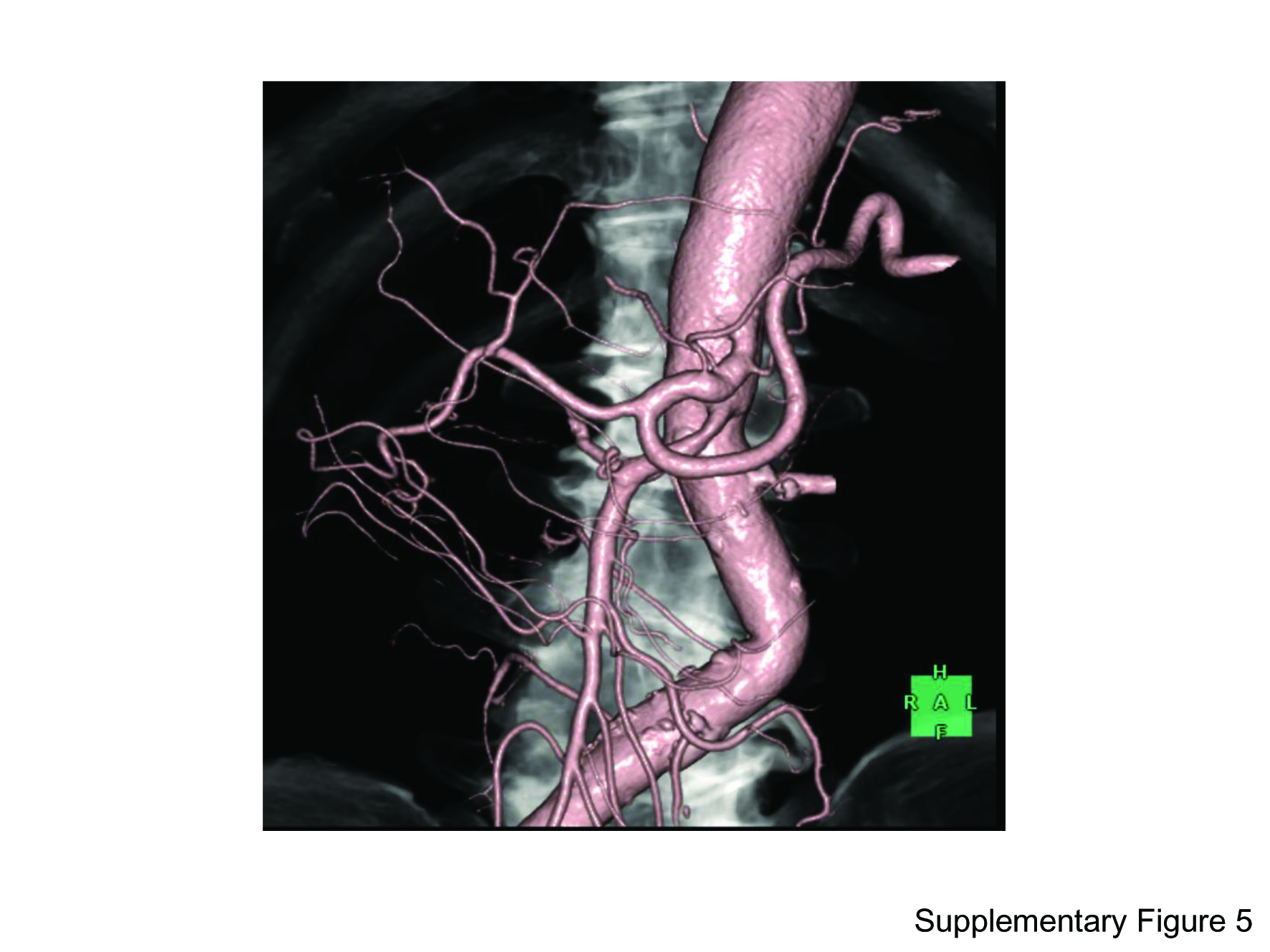

Supplement: Supplementary file 5 — Figure S5 Marked meandering of the abdominal aorta depicted using 3D‐CT images. [file JGH3-5-1041-s006.tif]
