# Supplementary material for: Safety, feasibility, and comfort of hepatic angiography and transarterial intervention with radial access for hepatocellular carcinoma
Source: JGH Open. 2021 Jul 30;5(9):1041–6. doi: 10.1002/jgh3.12628 (PMC8454468; doi:10.1002/jgh3.12628)
Supplement: Supplementary file 6 — Appendix S1: Supporting information. [file JGH3-5-1041-s002.docx]

**Supplementary text**

1. Protocol of computed tomography during arterial portography (CTAP)

About 50 mL (250mgI/kg) of contrast medium (Iopamiron 300; Bayer Yakuhin, Osaka, Japan) diluted with saline at 1: 2 ratio by volume was administered using an automatic power injector at a rate of 2.5 mL/s during CT scanning with 30 seconds delay from the initiation of contrast injection.

2. Protocol of computed tomography during hepatic arteriography (CTHA)

About 20 mL (100mgI/kg) of contrast medium (Iopamiron 300; Bayer Yakuhin, Osaka, Japan) diluted with saline at 1: 2 ratio by volume was administered using an automatic power injector at a rate of 2 mL/s during CT scanning with 9 seconds delay from the initiation of contrast injection.

3. Protocol of transarterial chemoembolization (TACE)

An anticancer agent, epirubicin (Farmorubicin; Nihon Kayaku, Tokyo, Japan) was mixed with iodized oil (Lipiodol Ultra-Fluid; Guerbet, Villepinte, France) at 1: 2 ratio by volume and then injected into the target arteries. Alternatively, 50 mg of cisplatin powder (IA-call; Nihon Kayaku) was solved in 5mL of lipiodol and then injected into the target arteries. An anticancer agent with lipiodol was infused until the braches of portal vein was depicted.

Embolization was then performed using gelatin sponge particles (Gelpart; Nihon Kayaku). Gelatin sponge particles were injected until the stagnation of blood flow of the feeding artery.
